# Protective Roles of Zinc and Selenium Against Oxidative Stress in Brain Endothelial Cells Under Shear Stress

**DOI:** 10.3390/antiox14040451

**Published:** 2025-04-09

**Authors:** Jacopo J. V. Branca, Massimo Gulisano, Alessandra Pacini

**Affiliations:** Department Experimental and Clinical Medicine, Anatomy and Histology Section, University of Firenze, L.go Brambilla 3, 50134 Firenze, Italy; jacopojuniovalerio.branca@unifi.it (J.J.V.B.); massimo.gulisano@unifi.it (M.G.)

**Keywords:** hypertension, millifluidic system, oxidative stress, NRF2, zinc, selenium, ZO-1, HBEC5i, brain endothelial cells

## Abstract

Background: Hypertension is a major risk factor for cerebrovascular diseases due to its damaging effects on the blood–brain barrier (BBB) and associated pathologies. Oxidative stress-induced endothelial damage plays a critical role in BBB disruption, potentially leading to cognitive impairment and neurodegeneration. In this study, we investigated the protective effects of two essential trace elements, zinc (Zn) and selenium (Se), against oxidative stress in human brain endothelial cells (HBCE5i) exposed to hypertensive shear stress. Using an innovative millifluidic system (LiveBox2), which allows for the precise simulation of continuous flow conditions, we replicated the hemodynamic forces associated with hypertension. Methods: Cells were treated with ZnCl_2_ (5–50 µM) or Na_2_SeO_3_ (50–500 nM) at concentrations selected based on previous studies and confirmed by cytotoxicity assays. Results: Our results demonstrated that shear stress significantly altered the localization of the tight junction protein zonula occludens-1 (ZO-1) and induced the nuclear translocation of the transcription factor NRF2, a hallmark of oxidative stress. Importantly, treatment with 10 µM ZnCl_2_ preserved ZO-1 membrane localization and prevented NRF2 translocation, as confirmed by quantitative image analysis. In contrast, Na_2_SeO_3_ did not provide comparable protection, although modest improvements in ZO-1 localization were observed in some replicates. Discussion: We discuss potential reasons for selenium’s limited efficacy, including differences in bioavailability and cellular uptake. Our findings underscore zinc’s promising role as a neurovascular protector and suggest that further investigation into more complex in vitro models and in vivo studies is warranted.

## 1. Introduction

Hypertension is a pervasive global health concern and a key modifiable risk factor for cerebrovascular disorders, including stroke, vascular dementia, and Alzheimer’s disease. Elevated blood pressure is associated not only with direct damage to the heart and kidneys but also with profound effects on the brain [1,2]. Chronic hypertension disrupts cerebral autoregulation, leading to ischemia and hypoxia that eventually result in neurodegeneration and cognitive decline [3]. A central component in the pathogenesis of hypertension-related brain damage is the impairment of the blood–brain barrier (BBB), a highly selective interface that protects the central nervous system (CNS) from harmful substances while regulating nutrient transport. The BBB is primarily formed by brain endothelial cells, which are tightly interconnected by complexes of tight junction (TJ) proteins, including zonula occludens-1 (ZO-1), occludin, and claudins. In an intact BBB, these proteins maintain low paracellular permeability and ensure neuronal homeostasis. However, under hypertensive conditions, mechanical forces and elevated shear stress can disrupt TJ organization, leading to increased BBB permeability and facilitating the entry of neurotoxic molecules [3,4]. Oxidative stress, characterized by the overproduction of reactive oxygen species (ROS), is recognized as a critical mediator of BBB breakdown. ROS can damage cellular structures and modulate signaling pathways that ultimately lead to TJ disassembly and endothelial dysfunction [5,6].

In hypertensive conditions, oxidative stress, characterized by excessive oxygen species (ROS) production, plays a crucial role in disrupting BBB integrity by damaging tight junction (TJ) proteins, such as ZO-1 [7].

Trace elements such as zinc (Zn) and selenium (Se) have emerged as important modulators of oxidative stress. Zinc is well known for its antioxidant properties; it acts as a cofactor for numerous enzymes, modulates the activity of NADPH oxidase, and stabilizes membrane proteins, including those that constitute tight junctions [8,9]. Several studies have demonstrated that zinc supplementation can mitigate oxidative damage in various tissues, including vascular endothelium [10,11,12]. Selenium, on the other hand, is incorporated into selenoproteins such as glutathione peroxidases, which play a central role in neutralizing ROS and maintaining redox balance [13,14]. Despite these well-documented antioxidant properties, the comparative efficacy of Zn and Se in protecting the BBB under hypertensive conditions remains insufficiently explored.

A novel aspect of our study is the application of the LiveBox2 (IVTech S.r.l., Pisa, Italy) millifluidic system, which provides a continuous, precisely controlled flow that better mimics the dynamic hemodynamic environment of brain endothelial cells in vivo. Unlike static or intermittent flow systems, the LiveBox2 permits the simulation of hypertensive shear stress conditions, thereby offering more physiologically relevant insights into the cellular responses to elevated flow [15,16]. This model allows us to assess not only the morphological changes in tight junction proteins but also to quantify oxidative stress markers such as the nuclear translocation of NRF, a key transcription factor activated in response to oxidative insult.

The present study aims to extend our understanding of the protective roles of zinc and selenium on BBB integrity. By using an in vitro model of HBEC5i cells subjected to controlled shear stress, we evaluate the effects of Zn and Se on the preservation of ZO-1 localization and the mitigation of oxidative stress, as evidenced by NRF2 translocation.

In summary, this work seeks to provide a comprehensive analysis of how zinc and selenium modulate the cellular responses of brain endothelial cells under hypertensive conditions, potentially offering novel insights into therapeutic strategies for preventing BBB dysfunction in hypertension and related neurovascular disorders.

## 2. Materials and Methods

### 2.1. Cell Line and Treatments

Human brain endothelial cells (HBEC5i) were obtained from ATCC (Milan, Italy), and cultured in a 1:1 mixture of DMEM/HAM’s F12, supplemented with 10% FBS (Fetal Bovine Serum), 1% antibiotics (penicillin and streptomycin, Euroclone, Milan, Italy), and EGM (Endothelial Growth Medium (% for 500 mL), consisting of hydrocortisone (0.02% 0.2 mL), bFGF (2% 2 mL), VEGF (Vascular Endothelial Growth Factor, 0.1% 0.5 mL), R3-IGF1 (Insulin-like Growth Factor 1, 0.1% 0.5 mL), ascorbic acid (0.1% 0.5 mL), hEGF (human Endothelial Growth Factor, 0.1% 0.5 mL), and GA-1000 (gentamicin sulfate–amphotericin, 0.1% 0.5 mL), and heparin (0.1% 0.5 mL) (Lonza, Euroclone, Milan, Italy), in a 5% CO_2_ humidified atmosphere at 37 °C. Experiments were performed on cells grown to approximately 80% confluence, which was chosen to facilitate a clear visualization of individual cell boundaries and an accurate quantification of intracellular fluorescence.

The concentrations of ZnCl_2_ (5 to 50 µM) and Na_2_SeO_3_ (50 to 500 nM) (Sigma Aldrich, Milan, Italy) were chosen based on previous studies that have investigated their physiological and protective roles in endothelial cells under oxidative conditions [17,18]. Similarly, Na_2_SeO_3_ concentrations within the selected range have been shown to enhance selenoprotein activity and effectively modulate oxidative stress responses in vitro without inducing toxicity [13,14]. These ranges were further confirmed through preliminary MTT assays in our laboratory, ensuring that the doses were optimal for our experimental conditions.

### 2.2. MTT Assay

Cell viability was assessed after 24 h of treatment with varying concentrations of the antioxidant substances. Briefly, cells were seeded in 96 well plates (25,000 cells/well) and allowed to attach for 24 h. The day after, the complete growth medium was replaced with starvation medium (complete growth medium without FBS and the EGM supplement) to avoid interference with the antioxidant treatments. Cells were then treated with varying concentrations of ZnCl_2_ (5 to 50 µM) or Na_2_SeO_3_ (50 to 500 nM) for 24 h. Following treatment, the medium was replaced with an MTT solution (1 mg/mL in DMEM without phenol red) and incubated for at least 20 min at 37 °C in a 5% CO_2_ atmosphere. The resulting formazan crystals were dissolved in DMSO, and absorbance was measured at 595 nm using a spectrophotometer (Multiscan DCF3000, ThermoFischer Scientific, Milan, Italy). Each experimental condition was performed in quintuplicate, and experiments were repeated three times.

### 2.3. Millifluidic Model

The LiveBox2 (LB2) system was used to generate a continuous, controlled flow ranging from 0 to 500 µL/min to simulate hypertensive shear stress conditions. Cells were seeded on round cover slips (Ø20 mm) in the LB2 chamber and allowed to reach 80% confluence. On the day of the experiment, cells were subjected to the designated flow rates for 2 h at 37 °C.

The LiveBox2 system was chosen for its ability to replicate physiological shear stress conditions in a dynamic environment, closely mimicking in vivo conditions. Unlike static culture models, which fail to simulate the mechanical forces experienced by endothelial cells, the LiveBox2 system provides continuous fluid flow, enhancing the physiological relevance of oxidative stress studies. Compared to other shear stress models, LiveBox2 offers greater control over flow parameters, allowing for a precise modulation of hypertensive-like conditions. Additionally, it enables long-term culture under controlled conditions, making it an ideal platform for studying endothelial responses to oxidative stress in a more realistic microenvironment [19,20].

### 2.4. Immunofluorescence Staining

After exposure to shear stress, cells were immediately fixed with 1% paraformaldehyde for 10 min at room temperature. Following fixation, cells were permeabilized with 0.1% Triton X-100 in PBS for 10 min and then blocked with 1% bovine serum albumin (BSA) in PBS for 30 min at room temperature. Primary antibody against NRF2 (diluted 1:100; Santa Cruz Biotechnology, Santa Cruz, CA, USA) and ZO-1 (diluted 1:50; Invitrogen, Milan, Italy) were applied overnight at 4 °C in blocking solution. The next day, cells were incubated with appropriate AlexaFuor-conjugated secondary antibodies (AlexaFluor 488 anti-mouse for NRF2, and AlexaFluor 568 anti-rabbit for ZO-1, Invitrogen, Milan, Italy). Nuclei were counterstained with DAPI (diluted 1:2000 for 5 min at room temperature). After washing with PBS, coverslips were mounted with anti-fade mounting medium.

### 2.5. Quantitative Image Analysis

**Figure 1:**
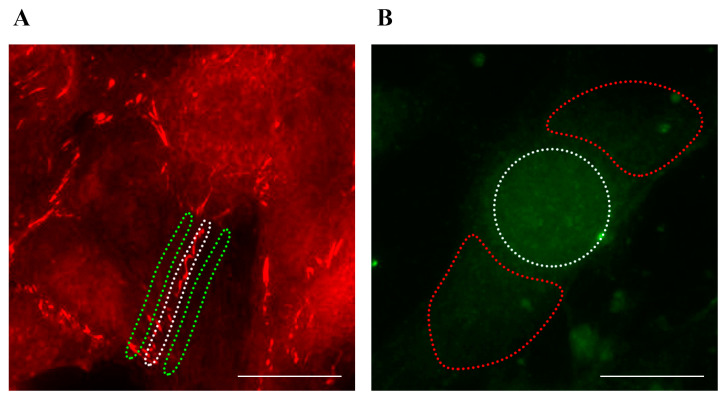
ROIs for FIJI analyses. The dotted areas indicate the ROIs selected for ZO-1 ((**A**), white dotted area for plasma membrane, green dotted area for adjacent cytoplasmic area) and NRF2 ((**B**), white dotted area for nuclear region, red dotted area for cytoplasmic region) analyses. Total magnification: 200×. Scale bar: 25 µm.

Images were analyzed using FIJI software (version 2.14.0/1.54f). For ZO-1, a region of interest (ROI) was drawn along the plasma membrane and another in the adjacent cytoplasmic area (Figure 1). The membrane/cytoplasm ratio of integrated intensity was calculated for each cell. A significant change in plasma membrane localization was defined as a ≥20% increase in the ratio relative to static controls. For NRF2, the nuclear and cytoplasmic intensities were measured using segmented ROIs. A cell was classified as showing NRF2 translocation if the nuclear intensity exceeded the cytoplasmic intensity by at least 1.5-fold. At least 15 fields (5 fields per replicate, with 3 independent replicates) were analyzed, and the percentage of cells with NRF2 nuclear translocation was calculated. Detailed statistical analysis, including summary graphs comparing the data distributions (violin plots), mean ± S.E.M., and significance (*p*-values), is provided in the figures.

### 2.6. Statistical Analysis

Data are expressed as mean ± S.E.M (Standard Error of the Mean).

To compare two independent groups (treated vs. untreated), an independent two-sample *t*-test was performed for normally distributed data, while the Mann–Whitney U test was used for non-normally distributed data. Assumptions for parametric tests were validated using the Shapiro–Wilk test for normality and Levene’s test for homogeneity of variance, with results confirming the appropriateness of the chosen statistical models. Statistical significance was set at *p* < 0.05.

## 3. Results

### 3.1. Shear Stress Induces TJ Disruption in HBEC5i Cell Line

**Figure 2:**
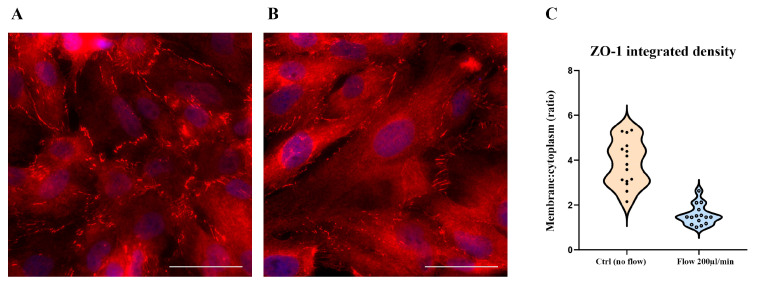
ZO1 localization in HBEC5i under different flow conditions. Human brain endothelial cells (HBEC5i) were cultured in the LiveBox2 system and subjected to a controlled flow rate ranging from 0 μL/min (no flow) to 200 µL/min. In the absence of flow (**A**), ZO1 (red) was properly localized at the cell periphery, ensuring tight junction integrity. Under shear stress conditions (200 µL/min, (**B**)), ZO1 exhibited an altered distribution, appearing stretched and disorganized. Nuclei were counterstained with DAPI (blue). The comparison of data distribution for each flow condition is reported as a violin plot (**C**). Control (no flow) 3.87 ± 0.16, 200 µL/min (flow) 1.55 ± 0.16, *p* < 0.05. Quantitative analysis revealed that the membrane/cytoplasm fluorescence intensity ratio of ZO1 decreased by approximately 60% in shear-stressed cells compared to controls (*p* < 0.05). The experiment was conducted three times, with five fields captured per experimental condition using a DCF350FX camera. Total magnification: 200×. Scale bar: 50 µm.

Under static conditions (0 µL/min), immunofluorescence revealed that ZO1 was uniformly localized at the plasma membrane of HBEC5i cells, indicating intact tight junctions (Figure 2A). However, when cells were exposed to a continuous flow of 200 µL/min, a marked redistribution of ZO-1 was observed. The protein appeared stretched and disorganized, with reduced intensity at the cell periphery (Figure 2B). Quantitative analysis demonstrated that the membrane/cytoplasm fluorescence intensity ratio of ZO-1 decreased by approximately 60% (*p* < 0.05) in shear-stressed cells compared to controls, confirming altered plasma membrane localization. A data distribution comparison is reported in Figure 2C.

### 3.2. Shear Stress-Induced NRF2 Nuclear Translocation

To assess whether shear stress triggers oxidative stress in HBEC5i cells, we examined the nuclear translocation of NRF2, a key transcription factor involved in cellular antioxidant defense mechanisms. The NRF2 translocates to the nucleus upon oxidative insult to activate cytoprotective genes [21,22,23].

**Figure 3:**
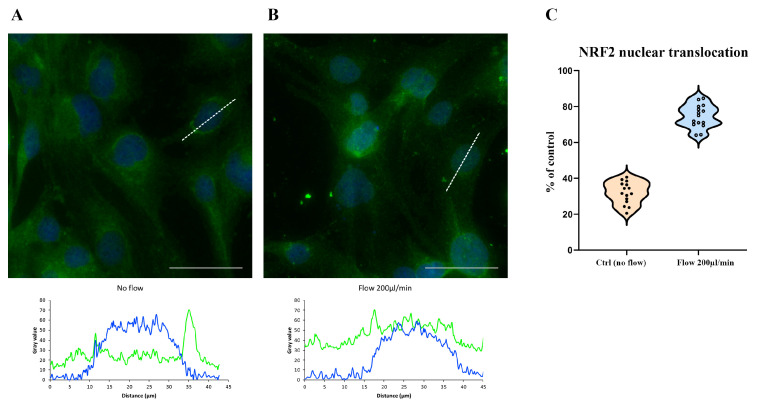
NRF2 nuclear translocation in HBEC5i cells under shear stress conditions. Human brain endothelial cells (HBEC5i) were cultured in the LiveBox2 system, which allows for a precise regulation of flow rates from 0 µL/min (no flow) to 200 µL/min. In (**A**), cells maintained under static conditions (0 µL/min) exhibited cytoplasmic NRF2 localization (green), as confirmed by the plot profile (DAPI-nucleus: blue line; NRF2 signal: green line). In contrast, exposure to a flow rate of 200 µL/min (**B**) induced NRF2 nuclear translocation, as illustrated by the corresponding plot profile. The comparison of data distribution for each flow condition is reported as a violin plot (**C**). Quantitative analysis revealed that the percentage of cells exhibiting NRF2 nuclear localization increased from 31.86% ± 1.34 under static conditions to 74.53% ± 2.76 under shear stress (*p* < 0.05). Nuclei were counterstained with DAPI (blue). The experiments were conducted three times, capturing five fields for each experimental condition using a DCF350FX camera. Total magnification: 200×. Scale bar: 50 µm.

Under static conditions, NRF2 was predominantly localized in the cytoplasm (Figure 3A). In contrast, cells exposed to 200 µL/min shear stress exhibited a significant nuclear translocation of NRF2, as evidenced by both qualitative imaging (Figure 3B) and quantitative analysis. Quantification revealed that the percentage of cells with NRF2 nuclear translocation increased from 31.86% ± 1.38 in controls (no flow) to 74.53% ± 2.87 in shear-stressed cells (*p* < 0.05). The criteria for classifying NRF2 translocation were based on a nuclear/cytoplasmic intensity ratio of ≥1.5. A data distribution comparison is reported in Figure 3C.

### 3.3. Zinc and Selenium Treatments Do Not Affect HBEC5i Viability

**Figure 4:**
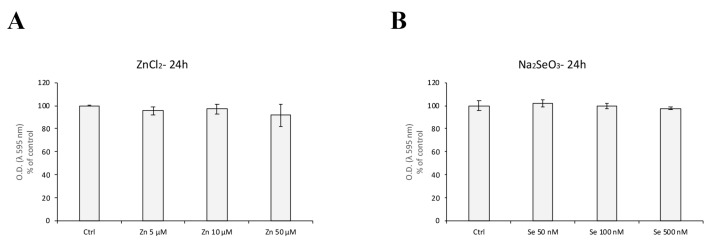
Cell viability assay of HBEC5i cells exposed to ZnCl2 or NaSeO_3_ for 24 h. HBEC5i cells were treated with increasing concentrations of ZnCl_2_ (5–50 µM) (**A**) and Na_2_SeO_3_ (50–500 nM) (**B**). No significant decrease in cell viability was observed compared to the untreated control cells (*p* > 0.05). Results are reported as mean ± S.E.M. (Standard Error of the Mean). The experiments were performed three times, with each experimental point conducted in quintuplicate.

MTT assays demonstrated that treatment with ZnCl_2_ (5–50 µM) and Na_2_SeO_3_ (50–500 μM) for 24 h did not significantly reduce cell viability compared to untreated controls (Figure 4). Based on these results, 10 µM ZnCl_2_ and 100 nM Na_2_SeO_3_ were used in subsequent experiments to evaluate their protective effects against shear-stress-induced oxidative damage.

### 3.4. ZnCl_2_ Prevents Oxidative Stress and TJ Disruption, While Na_2_SeO_3_ Does Not

**Figure 5:**
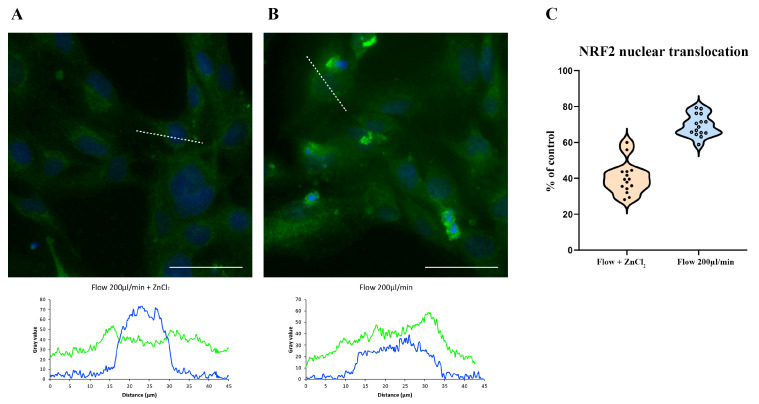
HBEC5i cells were subjected to a flow rate of 200 µL/min in the presence of 10 µM ZnCl_2_. As shown in (**A**), NRF2 (green) remained localized in the cytoplasm, indicating that oxidative stress was mitigated. The corresponding graph in (**B**) illustrates a profile comparison between cells exposed to shear stress with and without ZnCl_2_ treatment, revealing a statistically significant reduction in NRF2 nuclear translocation in ZnCl_2_-treated cells. Nuclei were counterstained with DAPI (blue). The comparison of data distribution for each flow condition is reported as a violin plot (**C**). ZnCl_2_ 40.08% ± 2.19, 200 µL/min (flow) 69.45% ± 2.03, *p* < 0.05. The experiments were performed three times, capturing five fields per experimental condition using a DCF350FX camera. Total magnification: 200×. Scale bar: 50 µm.

**Figure 6:**
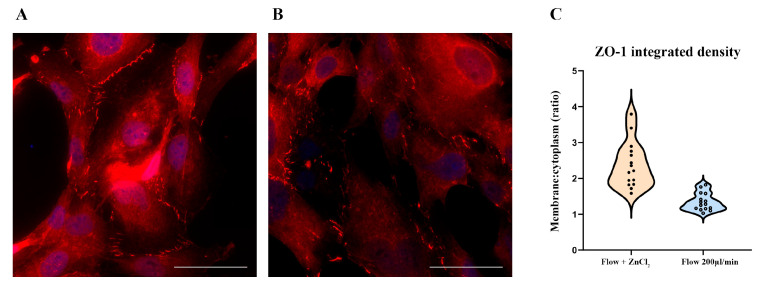
ZnCl_2_ (10 µM) maintains proper ZO-1 under shear stress conditions. HBEC5i cells were exposed to a 200 µL/min flow rate in the presence of 10 µM ZnCl_2_. In (**A**), ZO1 protein (red) remained correctly localized at the cell periphery, preserving tight junction integrity, whereas in (**B**), cells exposed to shear stress without ZnCl_2_ exhibited disrupted ZO-1 localization. The comparison of data distribution for each flow condition is reported as a violin plot (**C**). ZnCl_2_ 2.37 ± 0.15, 200 µL/min (flow) 1.34 ± 0.08, *p* < 0.05. Quantitative analysis revealed a statistically significant increase in the membrane/cytoplasm fluorescence intensity ratio of ZO-1 in ZnCl_2_-treated cells compared to untreated cells. Nuclei were counterstained with DAPI (blue). The experiments were performed three times, capturing five fields per experimental condition using DCF350FX camera. Total magnification: 200×. Scale bar: 50 µm.

When HBEC5i cells were treated with 10 µM ZnCl_2_ during exposure to a shear stress of 200 μL/min, immunofluorescence analysis showed that NRF2 largely remains in the cytoplasm (Figure 5A) and ZO-1 maintains its typical plasma membrane localization (Figure 6A). Quantitative image analysis confirmed that ZnCl_2_ treatment significantly increased the membrane/cytoplasm ratio of ZO-1 and reduced the percentage of cells exhibiting NRF2 nuclear translocation (*p* < 0.05) compared to shear-stressed cells without treatment (Figure 5B and Figure 6B).

**Figure 7:**
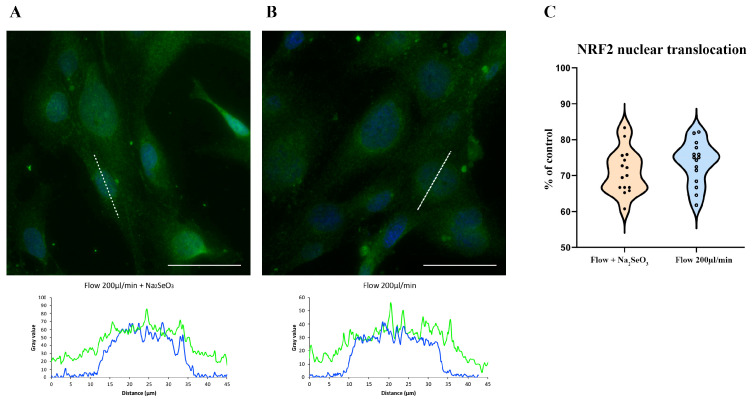
Na_2_SeO_3_ (100 nM) fails to prevent NRF2 nuclear translocation under shear stress. HBEC5i cells were subjected to a 200 µL/min flow rate in the presence of 100 nM Na_2_SeO_3_ (**A**). NRF2 (green) translocated to the nucleus, indicating oxidative stress induction, similar to cells exposed to shear stress without Na_2_SeO_3_ (**B**). The comparison of data distribution for each flow condition is reported as a violin plot (**C**). Na_2_SeO_3_ 71.08% ± 2.35, 200 µL/min (flow) 73.47% ± 2.37, not significant. Quantitative analysis revealed no significant difference in the percentage of cells exhibiting NRF2 nuclear translocation between Na_2_SeO_3_-treated and untreated shear-stressed cells. Nuclei were counterstained with DAPI (blue). The experiments were conducted three times, capturing five fields per experimental condition using a DCF350FX camera. Total magnification: 200×. Scale bar: 50 µm.

**Figure 8:**
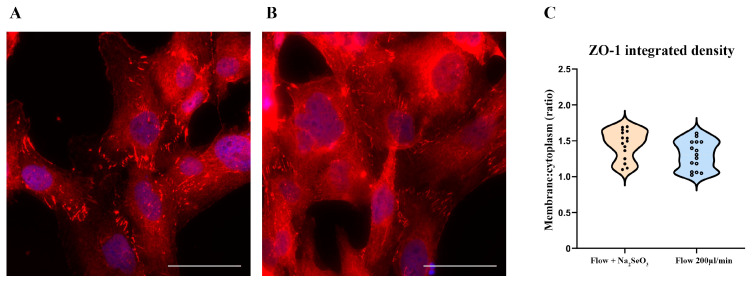
*Na_2_SeO_3_ (100 nM) treatment does not maintain ZO-1 localization under shear stress conditions.* HBEC5i cells were subjected to a 200 µL/min flow rate in the presence of 100 nM Na_2_SeO_3_. In (**A**), ZO-1 (red) failed to maintain its perimetral localization, mirroring cells exposed to shear stress without Na_2_SeO_3_ (**B**). The comparison of data distribution for each flow condition is reported as a violin plot (**C**). Na_2_SeO_3_ 1.45 ± 0.08, 200 µL/min (flow) 1.30 ± 0.02, not significant. Quantitative analysis confirmed that there was no significant difference in the membrane/cytoplasm fluorescence intensity ratio of ZO-1 between Na2SeO3-treated and untreated shear-stressed cells. Nuclei were counterstained with DAPI (blue). The experiments were performed three times, capturing five fields per experimental condition using a DCF350FX camera. Total magnification: 200×. Scale bar: 50 µm.

In contrast, treatment with 100 mM Na_2_SeO_3_ did not prevent NRF2 nuclear translocation nor preserve proper ZO-1 localization (Figure 7A,B and Figure 8A,B).

Although a modest improvement in ZO-1 membrane localization was observed in some replicates, this effect did not reach statistical significance. These findings suggest that, under the acute conditions modeled in this study, selenium’s protective efficacy is limited compared to that of zinc. The data distribution comparison is reported in each Figure 7C and Figure 8C.

## 4. Discussion

This study investigated the protective roles of zinc and selenium in blood–brain barrier (BBB) integrity under hypertensive shear stress conditions using an in vitro HBEC5i model. Our results confirm that exposure to shear stress induces oxidative stress, as demonstrated by the disruption of zonula occludens-1 (ZO-1) localization and the nuclear translocation of NRF2. These findings underscore the importance of oxidative stress in mediating BBB dysfunction, a critical factor in the pathogenesis of hypertension-induced neurovascular damage.

A key strength of our experimental design is the use of the LiveBox2 millifluidic system, which offers continuous and precisely controlled flow conditions. Unlike traditional static culture systems or models with intermittent flow, the LiveBox2 replicates the dynamic hemodynamic environment of brain endothelial cells in vivo more faithfully. This continuous flow creates a stable shear stress that better mimics hypertensive conditions, leading to more reproducible and physiologically relevant responses [19,20]. As such, our findings regarding tight junction disruption and NRF2 translocation can be considered reflective of the in vivo situation under hypertensive stress.

Our results demonstrate that treatment with 10 µM ZnCl_2_ effectively protects against shear stress-induced oxidative damage. Quantitative analysis showed that zinc treatment preserved ZO-1 localization at the plasma membrane and significantly reduced the proportion of cells with NRF2 nuclear translocation. These protective effects are consistent with the known antioxidant properties of zinc, which include the inhibition of NADPH oxidase activity and the stabilization of membrane proteins [10,11,12].

In contrast, the protective effect of selenium, delivered as 100 nM Na_2_SeO_3_, was limited. Several factors could contribute to this discrepancy. One possible explanation for the limited efficacy of selenium in our experimental model is its bioavailability and cellular uptake under shear stress conditions. Previous studies have suggested that selenium uptake is highly dependent on cell type, culture conditions, and extracellular transport mechanisms [24]. The endothelial cells used in our model may exhibit a lower expression of selenium transporters, leading to reduced intracellular accumulation and subsequent antioxidant activity. Another possibility is that selenium primarily exerts its protective effects through its incorporation into selenoproteins, which regulate redox balance rather than providing immediate protection against oxidative stress. This indirect mechanism may require prolonged exposure or specific metabolic conditions to be fully effective, which might explain why its effects were not as pronounced as those of zinc in our short-term experimental setting [25]. Previous studies have demonstrated that selenium supplementation can significantly mitigate oxidative stress in endothelial and neuronal cells [26]. However, the magnitude of this effect varies depending on the experimental model, selenium formulation, and dosage. For example, studies using sodium selenite at higher concentrations (~1 μM) reported stronger antioxidant responses, suggesting a possible dose-dependent effect [26]. Additionally, differences in oxidative stress induction methods, such as the use of chemical oxidants versus shear stress, may further explain variations in Se efficacy across studies [27].

Future investigations should focus on optimizing selenium bioavailability, potentially by using alternative Se compounds with higher cellular uptake (e.g., selenomethionine). Further studies could also explore the time-dependent effects of selenium supplementation, as its protective mechanisms may require extended exposure to fully activate selenoprotein-mediated antioxidant pathways.

While our in vitro model using brain endothelial cells under shear stress provides valuable insights into oxidative stress responses, it does not fully replicate the complexity of the BBB in vivo. The BBB is a highly specialized structure composed of endothelial cells, pericytes, and astrocytes, all of which interact dynamically to regulate barrier function, oxidative stress responses, and neurovascular homeostasis [28]. By focusing solely on endothelial cells, our model lacks the contribution of pericytes and astrocytes, which have been shown to modulate antioxidant defenses and reinforce BBB integrity under physiological and pathological conditions [29].

Pericytes are known to play a critical role in stabilizing endothelial junctions and regulating oxidative stress via the secretion of neuroprotective factors such as angiopoietin-1 and platelet-derived growth factor (PDGF) [30]. Their absence in our model may have influenced the degree of ZO-1 disruption observed under shear stress, potentially underestimating the BBB’s in vivo resilience.

Astrocytes contribute to BBB maintenance by releasing glial-derived neurotrophic factors and antioxidants, which can influence NRF2 activation and redox homeostasis [31]. The exclusion of astrocytes from our model may limit the extrapolation of our results to in vivo conditions, where astrocyte–endothelial interactions could enhance the protective effects of zinc and selenium.

Given these limitations, our findings should be interpreted with caution when considering their direct application to in vivo BBB dynamics. The absence of pericytes and astrocytes may lead to an oversimplification of oxidative stress responses, and additional studies using co-culture models or organ-on-chip platforms are needed to better capture the intricate cellular interactions within the BBB. Future research should integrate multi-cellular BBB models to validate whether zinc and selenium exhibit comparable protective effects in a more physiologically relevant system.

While our findings underscore the robust role of zinc in mitigating shear stress-induced oxidative damage and BBB disruption, several challenges remain for clinical translation. First, dosage optimization is crucial because the therapeutic window for trace elements like zinc and selenium is narrow. Excessive zinc intake, for instance, may lead to adverse effects such as immune dysfunction or interference with the metabolism of other essential minerals [10], and inappropriate selenium dosing may cause toxicity or paradoxical pro-oxidant effects [14]. Long-term safety profiles and potential side effects require thorough evaluation in preclinical and clinical settings. Moreover, the oxidative stress mechanisms implicated in BBB dysfunction are also involved in a range of other conditions, including neurodegenerative diseases (e.g., Alzheimer’s and Parkinson’s disease), cardiovascular disorders, and chronic inflammatory states. Thus, optimized zinc supplementation strategies might have broader therapeutic applications beyond hypertension, potentially benefiting multiple pathologies characterized by oxidative damage.

Given their broad-spectrum antioxidant properties, zinc and selenium supplementation may also have potential applications in aging-related oxidative stress, immune system modulation, and inflammatory disorders. Investigating their systemic effects in larger-scale studies, including in vivo models and clinical trials, will be essential to validate their efficacy across diverse oxidative stress-related diseases.

In addition to the cellular analyses presented, we attempted orthogonal assays to further validate our findings. Preliminary experiments using redox-sensitive fluorescent dyes (e.g., DCFDA) confirmed increased ROS production under shear stress, although the variability of these assays limited their inclusion in the final analysis. Similarly, while changes in tight junction integrity were inferred from immunofluorescence studies, direct measurements of barrier function (e.g., transendothelial electrical resistance or permeability assays) were not performed in this study but are planned for future investigations.

Despite these limitations, our study provides compelling evidence that zinc is a promising neurovascular protector in the context of acute shear stress. Although selenium did not show comparable efficacy in this acute model, its role in chronic oxidative stress scenarios remains to be fully explored. Future research should focus on refining dosing regimens, further elucidating the molecular mechanisms underlying the differential effects of zinc and selenium, and validating these findings in more complex and physiologically relevant models.

## 5. Conclusions

Our findings demonstrate that zinc effectively protects brain endothelial cells from shear stress-induced oxidative damage by preserving tight junction integrity and preventing NRF2 nuclear translocation. In contrast, selenium exhibited limited efficacy under the acute conditions studied. These results underscore zinc’s potential as a therapeutic agent for mitigating BBB dysfunction in hypertensive states and possibly in other oxidative stress-related conditions. Further studies in more complex models and in vivo systems are warranted to fully elucidate the mechanisms and optimize dosing strategies for clinical translation.

## Data Availability

Data contained within the article.
